# Efficient adsorption and photocatalytic degradation of Congo red onto hydrothermally synthesized NiS nanoparticles

**DOI:** 10.1007/s11051-013-1475-y

**Published:** 2013-02-13

**Authors:** Hongxu Guo, Yingchang Ke, Dongfeng Wang, Kaili Lin, Ruxiang Shen, Jianhua Chen, Wen Weng

**Affiliations:** 1 Department of Chemistry & Environmental Science, Zhangzhou Normal University, Zhangzhou, 363000 People’s Republic of China; 2 Shanghai Institute of Ceramics, Chinese Academy of Sciences, 1295 Dingxi Road, Shanghai, 200050 People’s Republic of China

**Keywords:** NiS nanoparticles, Adsorption, Photocatalytic degradation, Congo red, Mechanism

## Abstract

NiS nanoparticles (NiS NPs) have been hydrothermally prepared and characterized by the methods of X-ray diffraction, scanning electronic microscope, X-ray photoelectron spectroscopy, UV–Vis diffuse reflectance spectra, photoluminescence, and thermogravimetric analysis. NiS NPs exhibit fast adsorption in the removal of Congo red (CR) in aqueous solution, in which the pseudo-second-order model was the best to describe the adsorption kinetics, and the intraparticle diffusion was not only the rate-limiting step. The NiS NPs also exhibit efficient photocatalytic activity in the degradation of CR under visible-light irradiation, in which the 30 mg/L CR was almost completely degraded after illumination during 210 min. The •OH radicals in the process of photocatalytic degradation were observed by fluorescence technique.

## Introduction

Colored dyes have been extensively applied in the textile, paper, cosmetics, pharmaceuticals, and food industries. Dye-contaminated water especially from the textile industry (Judd and Jefferson 2003) is difficult to clear due to objective causes. Most of those colored dyes are synthetic in nature and are usually composed of aromatic rings in their molecular structure, which makes them carcinogenic and mutagenic, inert, and non-biodegradable when discharged into wasters without appropriate treatment. Therefore, the removal of such colored agents from polluted waters is very urgent based on the point of human health and environmental resource protection (Allen et al. 2004; Özaca and Şengi 2004).

At the present time, there is a wide range of treatment technologies to remove several dyes from wastewaters. The conventional methods for the removal of dyes from aqueous solutions include precipitation, ion exchange, filtration, and electrochemical treatment (Rott and Minke 1999; Forgacs et al. 2004; Aksu 2005), which all have significant disadvantages such as incomplete color removal, high-energy requirements, and other waste products that require further disposal. Therefore, it is desirable to seek adequate method for the removal of toxic chemicals from wastewaters. Recently, advanced oxidation processes based on semiconductor photocatalysts have been the focus of much research for their wide applications in environmental purification in wastewater (Kudo et al. 1999; Ishikawa et al. 2002; Kim et al. 2004).

Among the various semiconductors, sulfide semiconductor with a wide band gap has been regarded as a promising photocatalyst, since it presents more activity in long band of visible light. Recently, many researchers have focused their attentions on the preparation and characterization of the novel sulfide semiconductor with effective photocatalytic activity. For example, hexagonal ZnS spheres self-assembled from ZnS nanocrystals exhibit enhanced photocatalytic activity for degradation of Rhodamine-B (Wu et al. 2009); AR 66 dye could be effectively decolorized by chitosan capped CdS composite nanoparticles under visible-light irradiation (Jiang et al. 2009); more recently, the low-temperature synthesis of uniform Sb_2_S_3_ nanorods with visible-light-driven photocatalytic activities has been reported (Han et al. 2010).

NiS nanoparticles(NPs), as a useful semiconductive material, has a number of applications in various fields such as IR detectors, electrode in PEC storage devices, solar storage, and hydrosulfurization catalysis (Mane and Lokhande 2000; Wong et al. 1992; Fernandez et al. 1993). However, up to date, very few researches on the photocatalytic activities of NiS nanocrystalline have been reported. Recently, Ejhieh and Khorsandi have reported the syntheses of NiS–P zeolite (Ejhieh and Khorsandi 2010) and NiS–clinoptilolite zeolite (Ejhieh and Khorsandi 2011) prepared by ion exchange and precipitation procedures, which possessed well photo-degradation properties under UV irradiation.

**Scheme 1:**
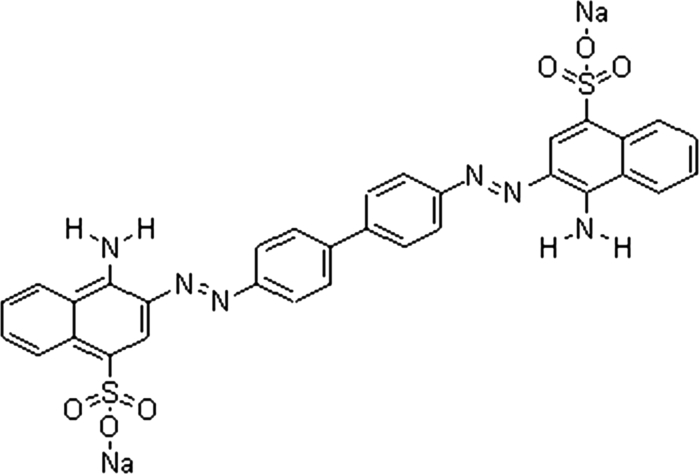
Molecule structure of Congo red

It has been demonstrated that the photocatalytic degradation rate depends greatly on the adsorption behavior between photocatalyst and pollutant molecules, and the adsorption of pollutant molecules on catalyst can significantly improve the degradation efficiency (Tryba et al. 2003; Zhao et al. 2004; Zhang et al. 2006). It is of great importance to investigate the adsorption process of organic pollutants on the catalyst surface to clarify the mechanism of photocatalytic reactions, which can facilitate their applications in contaminant destruction. Furthermore, the combination of adsorption and photocatalytic degradation may be a good choice to thoroughly treat organic pollutants in waters. In this study, the as-prepared NiS NPs has been prepared from hydrothermal synthesis and characterized by several means, such as X-ray diffraction (XRD), scanning electronic microscope (SEM), TEM, X-ray photoelectron spectroscopy (XPS), UV–Vis diffuse reflectance spectra (DRS), thermogravimetric analysis (TGA), and photoluminescence (PL) measurements. Furthermore, the adsorption behavior (including adsorption kinetics and adsorption isotherms) and photocatalytic degradation of Congo red (CR) (see Scheme 1) from aqueous solutions have been further studied in details.

## Experimental section

### Materials and preparation of NiS NPs

NiSO_4_, NaOH, and thioacetamide, purchased from Shanghai Chemical Reagent Corp., were used without further purification. Distilled deionized water with a resistivity of 18 MΩ/cm was used. The NiS NPs were prepared via typical hydrothermal method as follows: a mixture of NiSO_4_ (10 mmol), NaOH (15 mmol), thioacetamide (12 mmol), and distilled deionized water (60 mL) under magnetic stirring to form a homogeneous solution. The reaction mixture was then sealed in a 100-mL Teflon-lined reactor and heated in an oven at 160 °C for 24 h and then slowly cooled to room temperature naturally. After filtering the obtained powder was washed with water and ethanol several times to ensure elimination of the impurities adsorbed by the target product. Finally, the black NiS NPs powder was obtained after drying at 70 °C in a dust-proof environment.

### Analytical techniques for characterization of NiS NPs

The XRD patterns were tested on a Rigaku D/MAX-RB diffractometer with monochromatized CuKα radiation (*λ* = 1.5418 Å). The generator was operated at 40 kV and 25 mA. The samples were scanned at diffraction angle from 10 to 80° at the scanning rate of 0.068°/s. Morphological observations were performed on a field emission scanning electron microscope (FESEM, S-4800, Hitachi, Japan) and transmission electron microscopy(TEM) (JEOL, JEM-2000EX). XPS experiments were carried out in an ultrahigh vacuum using the ESCALAB Mark II X-ray photoelectron spectroscopy (XPS, VG Scientific, UK) with Mg Kα radiation (1253.6 eV) from the Mg anode source. The high-resolution scans of core level spectra were recorded with an energy step of 0.05 eV and set to 15 eV pass energy. The binding energy was referenced to at 284.6 eV to the C1s peak. Experimental data were deconvolved by Gaussian–Lorentzian mixture peak-fitting software. A PerkinElmer Lambda 35 UV/Vis spectrometer equipped with a labsphere diffuse reflectance accessory was used to characterize the reflectance spectra of the catalysts over a range of 200–800 nm. Labsphere RSA-PE-20 was employed as a reflectance standard. PL spectra were recorded with a Hitachi F-4500 spectrofluorometer at room temperature. TGA was performed on a TG 209 thermal analyzer at a heating rate of 10 °C/min, using an N_2_ atmosphere.

### Adsorptive kinetic and equilibrium experiments

All adsorption experiments were operated at room temperature (26 ± 0.5 °C). For the adsorption kinetics experiments, 0.10 g of NiS NPs powder was added to 200 mL of CR aqueous solution at the initial concentrations of 40, 60, and 80 mg/L. The suspensions were left in conical flasks and agitated in an isothermal reciprocating shaker at 150 rpm under room temperature. At appropriate time intervals, the aliquots were withdrawn from the solutions and centrifuged for 10 min at 6,000 rpm (rcf 604 g) to separate solid particles. The residual CR concentrations in the supernatant solutions were determined by absorbance measurement using UV–Visible spectrophotometer (UV/Vis spectrophotometer, WFZUV-2000) at its maximum absorption wavelength of 505 nm. It was then computed to CR concentration using standard calibration curve. The amount of CR adsorbed onto NiS powders at any time was computed by the difference between the initial CR concentration and the concentration after adsorption.

All batch equilibrium experiments were also carried out in conical flasks and rotated in the reciprocating shaker under the same conditions. To obtain the adsorption isotherm, the as-prepared NiS powders at a constant dose of 0.5 g/L were placed in CR solution of different concentrations (10–80 mg/L). Aliquots were taken from the flask at equilibrium, centrifuged, diluted, and analyzed by UV–Visible spectrophotometer to determine the initial and equilibrium CR concentrations in the solutions. The amount of CR adsorbed onto NiS NPs sample at equilibrium was calculated by the difference between the initial and equilibrium CR concentrations.

### Photocatalytic activities test

The photocatalytic degradation of CR under visible light was used to evaluate the photocatalytic activities of the NiS NPs samples. A 250 W indoor fluorescent lamp was used as the light source with a 420-nm cutoff filter to provide visible-light irradiation. The average light intensity striking the surface of the reaction solution was 25 mW/cm^2^. To test the photocatalytic degradation of CR, a solution containing a known initial concentration of CR and NiS NPs photocatalyst was allowed getting adsorption equilibrium for 120 min in the darkness. Then the solution was exposed to visible-light irradiation under magnetic stirring. At given time intervals, 4 mL of suspension was sampled and centrifuged to remove the photocatalyst powders. The concentration of the dye after photocatalytic degradation was determined with a spectrophotometer (UV/Vis spectrophotometer, WFZUV-2000) at *λ*_max_ = 505 nm and a calibration curve. Total organic carbon (TOC) of the solution was measured by a TOC Analyzer (Shimadzu TOV-V_CPH_).

## Results and discussion

### XRD spectrum of NiS NPs

**Fig. 1:**
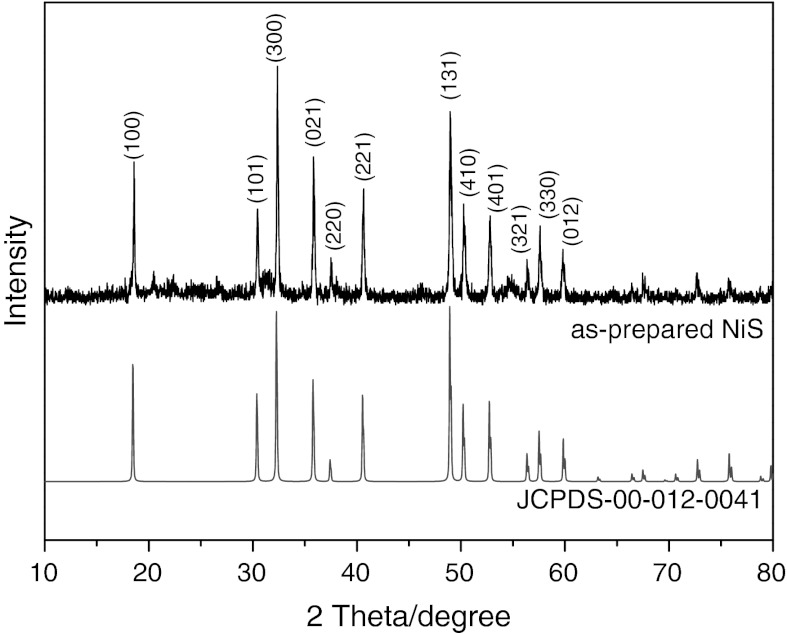
XRD patterns of the as-prepared NiS sample

Figure 1 shows the XRD patterns of the as-prepared nanostructured NiS samples. All peaks can be well indexed to rhombohedral structured NiS with space group of *R*3*m* and the cell parameters *a* = 9.61 Å, *c* = 3.16 Å, which shows a good agreement with the literature data (ICDD PDF No. 12-0041).

### SEM images of NiS NPs

**Fig. 2:**
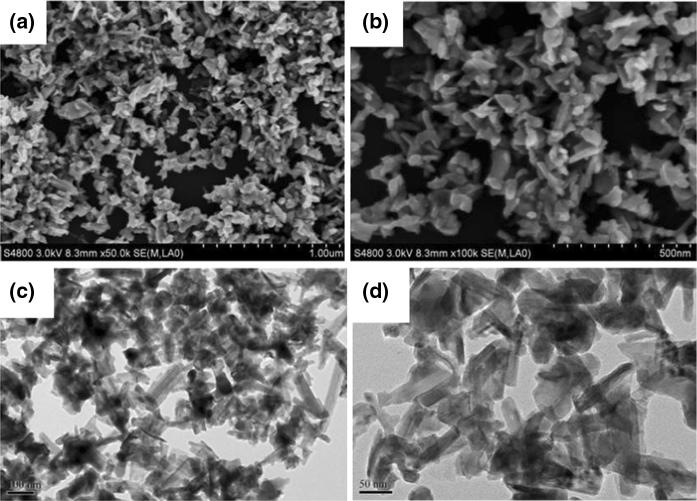
SEM (**a**, **b**) and TEM (**c**, **d**) images of NiS NPs

Figure 2 presents the SEM and TEM images of the prepared NiS nano-powders. The SEM images (Fig. 2a, b) show that the obtained sample consists of many monodisperse nanoparticles with blocky morphology. It can be also observed from the high magnification TEM images (Fig. 2c, d) that the particles were composed of blocky particles with the width sizes of 20–50 nm and the length sizes of 80–150 nm.

### XPS spectrum of NiS NPs

**Fig. 3:**
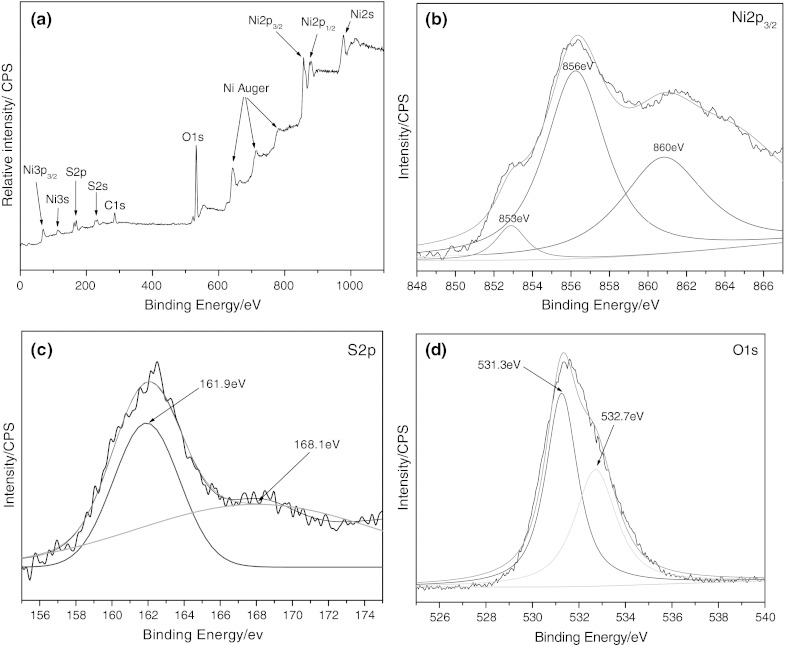
High-resolution XPS spectra of **a** survey, **b** Ni 2p_3/2_, **c** S2p, and **d** O1s

X-ray photoelectron spectroscopy (XPS) data of the as-prepared sample are shown in Fig. 3 to analyze the surface chemical states. The C1s core levels of sample showed one peak located at binding energy 287 eV, corresponding to the adventitiously polluted carbon.

The survey scan spectrum (Fig. 3a) indicates the presence of Ni, S, and O in the sample. The peak in 853 and 860 eV (Fig. 3b) can be attributed to that of Ni^2+^ bonded to sulfur. The contribution at 856 eV can be interpreted as Ni^2+^ bonded to hydroxide. As shown in S2p spectrum (Fig. 3C), the S2p_3/2_ peak at 161.9 eV can be observed. The peak at 168.1 eV arises from a surface sulfate species, probably coming from the materials or surface oxidation product. The O1s spectrum (Fig. 3d) of the as-prepared NiS sample is fitted with two peaks, one at a binding energy of 531.3 eV and the second at 532.7 eV. The peak at 531.0 is attributed to a hydroxyl group chemisorbed to nickel at the NiS nanoparticle surface, and the peak at 532.7 eV can be considered to arise from H_2_O adsorbed on the surface. The intensity of the hydroxyl peak is greater than that of the surface H_2_O peak, indicating that a greater proportion of surface oxygen is attributable to surface hydroxyl nickel complex.

### UV–Vis diffuse reflection spectrum of NiS NPs

**Fig. 4:**
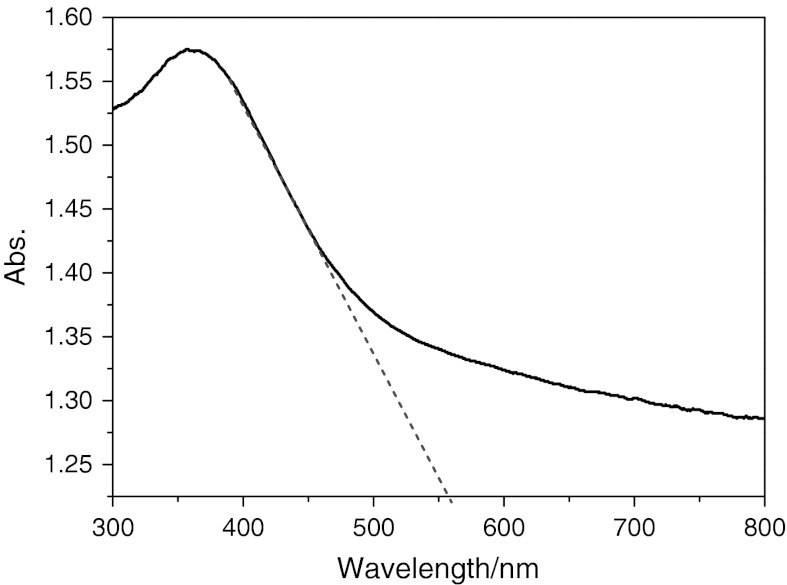
UV–Vis diffuse reflectance spectrum of the nanoparticles NiS sample

The photo-absorption ability of the material was detected by the UV–Vis diffuse reflectance spectrum, as shown in Fig. 4. The NiS NPs showed the strong photo-absorption properties in the UV–Vis light region. The band gap energy (*E*_g_) of the material can be estimated by the formula: *E*_g_ = 1,240/*λ*_g_, where *λ*_g_ is the wavelength corresponding to the intersection point of the vertical and horizontal parts of the spectrum. From Fig. 4, the wavelength of the absorption edge of the as-prepared sample was 555 nm. Thus, the band gap energy estimated from the absorption edge was about 2.23 eV. This result indicates that the NiS NPs have a suitable band gap for photocatalytic decomposition of organic contaminants under visible-light irradiation.

### Fluorescence spectrum of NiS NPs

**Fig. 5:**
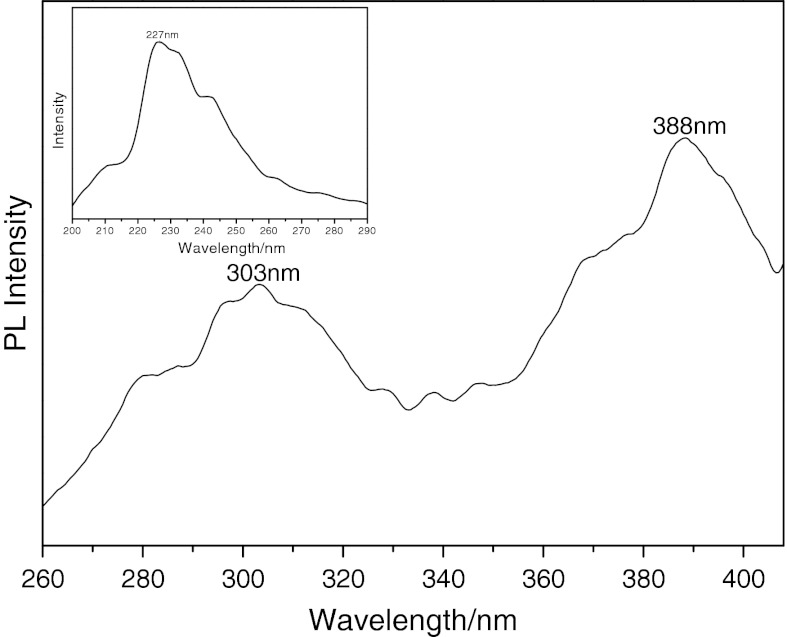
Fluorescene spectrum of the as-prepared NiS nanosheets measured at room temperature at an excitation wavelength of 227 nm (*inset*)

The optical properties of the products were further investigated. Figure 5 shows the fluorescence spectrum of NiS NPs under an excitation *λ*_ex_ = 227 nm (inset, Fig. 5). There is a broad emission for NiS NPs, and the top of the emission peak is separated into two peaks, locating at 303 and 388 nm, respectively. The separated peaks might be aroused by the defects in the interfacial region due to electronic transitions. The fluorescence emission peak at 388 nm could be attributed to a sulfur vacancies and interstitial sulfur lattice defects (Becher and Bard 1983). During the photoluminescence process, sulfur vacancies and defects can easily bind photo-induced electrons. Therefore, the fluorescence signals can easily occur.

### Thermal stability analyses of NiS NPs

**Fig. 6:**
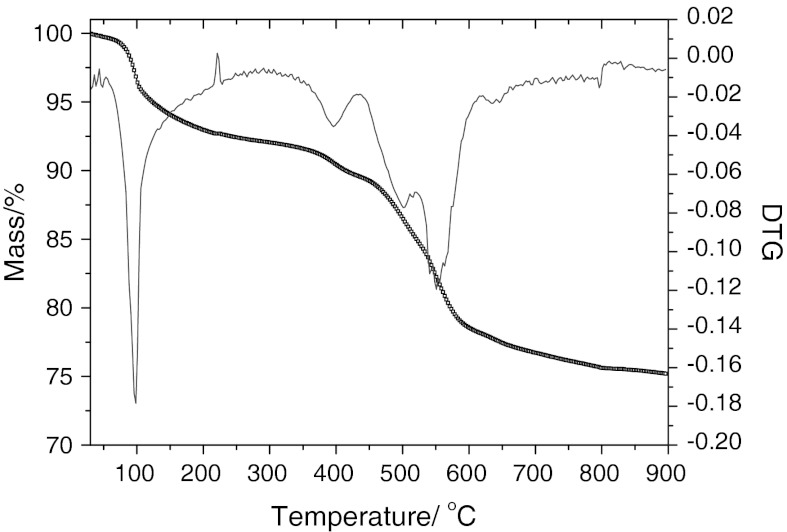
TG-DTA curve of the NiS nanoparticle sample

In order to explore the thermal stability of the hydrothermal precursor of NiS NPs powder in nitrogen atmosphere, the possible gravimetric and thermal changes were investigated by TGA and DTGA, as shown in Fig. 6. The TGA curve indicated that the increase in temperature resulted in weight loss (about 5.3 %). The weight loss region from 40 to 400 °C was caused by the loss of adsorbed water and hydroxide group. On the DTGA curve before 600 °C two exothermic and four endothermic peaks were observed. The endothermic peak at 100 °C was mainly attributed to the loss of the adsorbed water, while the three endothermic peaks from 400 to 550 °C may be attributed to the gradual loss of sulfur in the process of structural collapse.

### Studies on adsorption properties

**Fig. 7:**
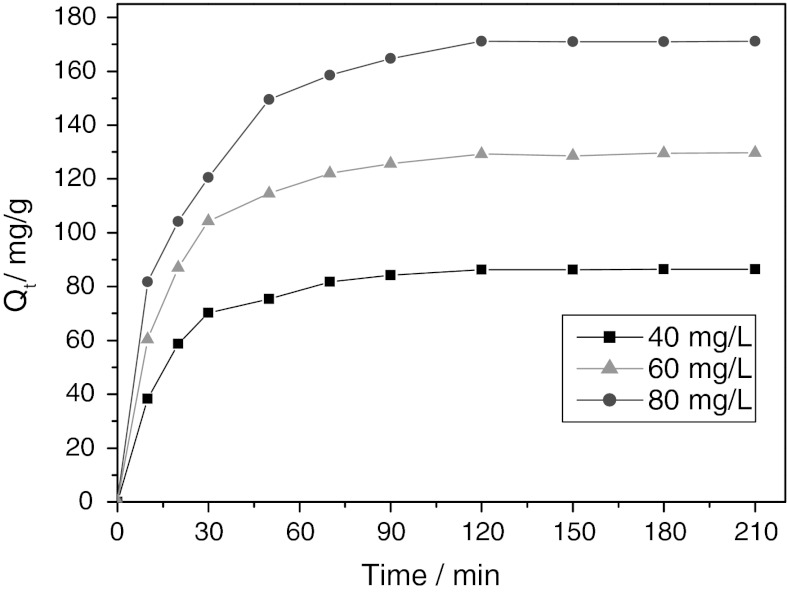
Plots of the variations of the amount of the adsorbed CR over contact time

To determine the time necessary to obtain equilibrium adsorption, the effect of contact time on adsorption of CR onto NiS NPs was studied at three different initial concentrations of 40, 60, and 80 mg/L. Fig. 7 showed the variations of the amount of the adsorbed CR over contact time. Obviously, CR adsorbed rapidly onto NiS NPs at the beginning. The adsorption equilibrium of CR was achieved after 120 min and no remarkable changes were observed for longer contact time.

It is meaningful to predict the rate at which contamination is removed from aqueous solution in order to design an adsorption treatment plant. In order to explicate the adsorption mechanism for the sorption of a CR dye onto nanostructure NiS from a liquid solution, two common kinetic models, namely the pseudo-first-order and pseudo-second-order model, were used to investigate the adsorptive rate. The pseudo-first-order model, is more suitable for lower concentrations of solution and can be expressed by the following linear form (Langergren and Svenska 1898):

1 $$ \ln (Q_{\text{e}} - Q_{\text{t}} ) = \ln Q_{\text{e}} - k_{1} t $$

where *Q*_e_ and *Q*_t_ (mg/g) are the amounts of adsorbed CR at equilibrium and at time *t*, respectively, *k*_1_ (1/min) presents pseudo-first-order rate constant, and *t* (min) is the time. Value of *k*_1_ in the linear form can be calculated from the slope of the plot of ln(*Q*_e_ − *Q*_t_) versus *t*.

The rate of the pseudo-second-order reaction is mainly dependent on the amount of solute adsorbed on the surface of adsorbent and the amount adsorbed at equilibrium. The pseudo-second-order model can be represented in the following linear form (Ho and McKay 1998):

2 $$ \frac{t}{{Q_{\text{t}} }} = \frac{1}{{k_{2} Q_{\text{e}}^{2} }} + \frac{t}{{Q_{\text{e}} }} $$

where *k*_2_ [g/(mg min)] is pseudo-second-order rate constant. Value of *k*_2_ and *Q*_e_ can be experimentally determined from the intercept and slope of the plot of *t*/*Q*_t_ versus *t*.

**Fig. 8:**
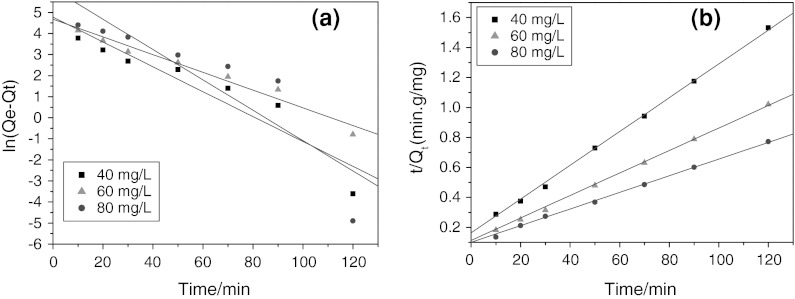
The linear plots of the pseudo-first-order (**a**) and pseudo-second-order (**b**) kinetic models at three initial concentrations

Figure 8 showed the linear plots of the pseudo-first-order and pseudo-second-order kinetic models at three initial concentrations of 40, 60, and 80 mg/L. The corresponding kinetic parameters obtained from the two models (*k*_1_, *k*_2_, *Q*_e1, cal,_ *Q*_e2, cal_, and *R* ^2^) were listed in Table 1. It can be found that the fitting of experimental data to the pseudo-first-order model was not so ideal, with rather low correlation coefficients of 0.9439, 0.9650, and 0.8887 for 40, 60, and 80 mg/L, respectively, indicating that the experimental data did not well obey the pseudo-first-order kinetic model. Differently, the linear plots of *t*/*Q*_t_ versus *t* with higher correlation coefficients (are close to unity) indicated that the adsorption kinetics of CR onto NiS NPs ideally obeyed the pseudo-second-order kinetic model. On the other hand, the same result can be also concluded from the deviation between the calculated and experimental *Q*_e_ values as listed in Table 1, that is, there were minor deviations between the calculated and experimental *Q*_e_ values for the pseudo-second-order model, while the calculated *Q*_e_ values for the pseudo-first-order model remarkably deviated the experimental *Q*_e_ values. Therefore, the pseudo-second-order kinetic model was much more accurate for CR adsorption process over NiS NPs in the solution.

**Table 1:** The determined constants of pseudo-first-order and pseudo-first-order kinetic model with correlation coefficients (*R* ^2^) at different initial concentrations

Initial (CR)	*Q*_e, exp_ (mg/g)	Pseudo-first-order kinetics model			Pseudo-second-order kinetics model		
		*k*_1_ (min^−1^)	*Q*_e,1_ (mg/g)	*R* ^2^	*k*_2_ (g/mg min)	*Q*_e,2_ (mg/g)	*R* ^2^
40 mg/L	87.05	0.0590	118.68	0.9439	7.77 × 10^−4^	88.73	0.9986
60 mg/L	130.03	0.0421	107.45	0.9650	5.59 × 10^−4^	133.33	0.9982
80 mg/L	172.38	0.0720	453.45	0.8887	2.72 × 10^−4^	180.18	0.9997

**Fig. 9:**
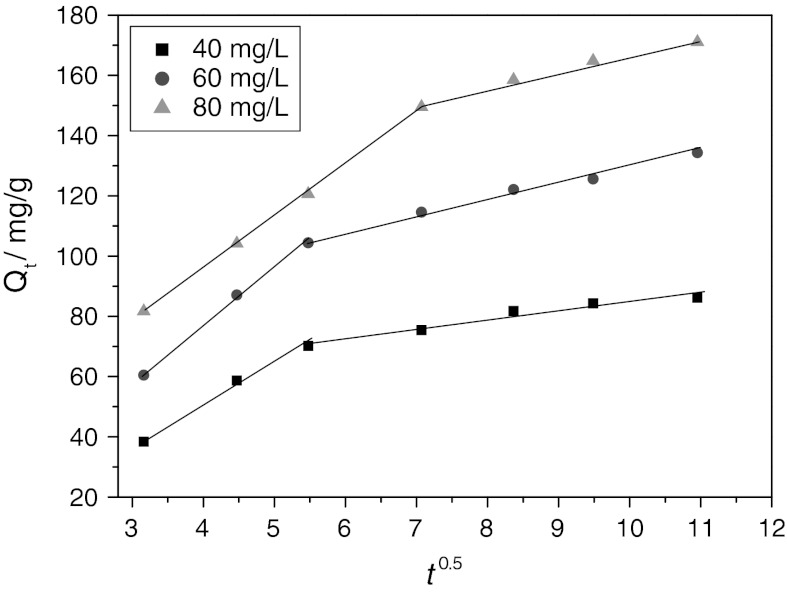
The linear plot of the portions of *Q*_t_ versus *t* ^0.5^ at three initial concentrations

In order to gain insight into the adsorption mechanism, the determination of the rate-limiting step in the adsorption process is required. The kinetic models mentioned above were unable to identify the diffusion mechanism, intraparticle diffusion model based on the theory proposed by Weber and Morris (1962) was then evaluated to further study the adsorption kinetic data. This model can be expressed as follows:

3 $$ Q_{\text{t}} = k_{\text{id}} t^{0.5} + C $$

where *k*_id_ [mg/(g min^0.5^)] is the intraparticle diffusion rate constant and *C* presents the boundary layer effect of the adsorption. Each plot of *Q*_t_ versus *t* ^0.5^ at different initial concentrations presents multi-linearity, indicating that two steps occurred in the adsorptive process, as illustrated in Fig. 9. The initial portion and the second portion in each plot may be produces in boundary layer effect and intraparticle diffusion, respectively. The initial steep-sloped portion, from 0 to 5 min^0.5^ for 40 and 60 mg/L while from 0 to 7 min^0.5^ for 80 mg/L, is attributed to external surface adsorption or instantaneous adsorption. The gentle-sloped portion followed by the initial portion can be attributed to gradual adsorption stage where intraparticle diffusion was rate-limiting (Wu et al. 2005).

**Fig. 10:**
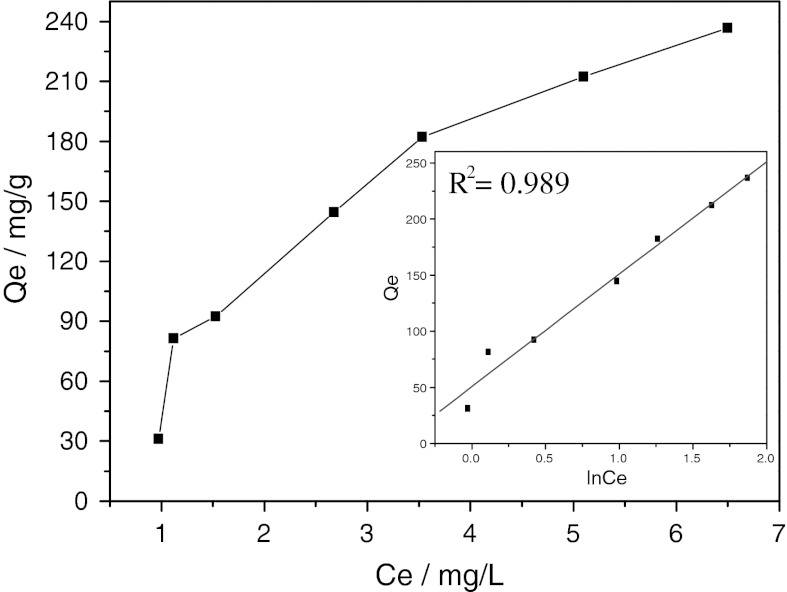
The adsorption isotherm of CR on NiS nanoparticles. *Inset* presents the linear plot for the Temkin model

Adsorption isotherm is an important means to study the adsorption and its mechanism. Figure 10 presents the equilibrium adsorption characteristics of CR onto NiS NPs at room temperature. It can be observed that the equilibrium adsorption capacity, *Q*_e_, increased with the increase of Ce. With the increase of initial CR concentration from 10 to 80 mg/L, the amount of the adsorbed CR at equilibrium increased from 31.34 to 236.83 mg/g. The explicit isotherm of L-shape, according to Giles et al. (1960), indicated that there is no strong competition between the solvent and the dye to occupy the NiS NPs.

To further describe the equilibrium adsorption isotherm, three common isotherm models, that is, the Langmuir isotherm, the Freundlich isotherm, and the Temkin isotherm, were used to analyze the equilibrium experimental data for the sorption of CR on NiS NPs.

The Langmuir equation is applicable to both physical and chemical adsorption; assuming a mono-molecular layer adsorption on a uniform surface while ignoring the lateral interactions between the adsorbed molecules (Özacar and Şengil 2005), its linear form of the Langmuir isotherm model is described by the following equation:

4 $$ \frac{1}{{Q_{\text{e}} }} = \frac{1}{{Q_{0} }} + \frac{1}{{Q_{0} b}} \cdot \frac{1}{Ce} $$

where *Q*_o_ (mg/g) and *b* (L/mg) are the Langmuir constants relating to adsorption capacity and rate of adsorption, respectively, *Q*_e_ is the amount of CR adsorbed at equilibrium (mg/g) and *Ce* is the liquid-phase equilibrium concentration (mg/L).

Freudlich equation is also applicable to both physical and chemical adsorption in heterogeneous systems (Özacar and Şengil 2003), which assume that the adsorption heat of a non-uniform surface logarithmically decreases with increasing surface coverage. The well-known logarithmic form of the Freundlich isotherm is expressed by the following equation:

5 $$ \ln Q_{\text{e}} = \ln K_{\text{F}} + \frac{1}{n}\ln Ce $$

where *Q*_e_ is the solid phase equilibrium concentration (mg/g), *Ce* presents the liquid-phase equilibrium concentration (mg/L), and *K*_F_ and n are Freundlich constants with *n* giving an indication of the facility with which the adsorption process takes place. *K*_F_ [(mg/g)(L/mg) 1/*n*] is the adsorption capacity of the adsorbent and represents the quantity of dye adsorbed onto the activated carbon per unit of equilibrium concentration.

The Temkin equation, however, is applicable to only chemical adsorption and assumes that the adsorption heat linearly decreases with increasing adsorption quantity, and the adsorption binding energy is distributed uniformly (Mall et al. 2005). The Temkin isotherm has been applied in the following form:

6 $$ Q_{\text{e}} = B\ln A + B\ln Ce $$

where *B* is the *RT*/*b*, *b* (J/mol) is the Temkin constant related to adsorption heat, *T* (K) is the absolute temperature, *R* [8.314 J/(mol K)] is the gas constant, and *A* (L/g) is the Temkin isotherm constant. *B* and *A* can be calculated from the slope and intercept of the plot of *Q*_e_ against ln*Ce*.

The experimental data on CR equilibrium adsorption onto NiS nanoparticles were fitted by the above-mentioned three isotherm models. The calculated adsorption parameters and correlation coefficients are summarized in Table 2, in which the experimental data were fitted much better with the Temkin isotherm than the other two isotherms. The correlation coefficient *R* ^2^ for the Temkin isotherm reached 0.989, which was apparent than that of Langmuir and Frundlich isotherms. Apparent adsorbent-adsorbate interaction on adsorption isotherms observed based on the Temkin isotherm was also consistent with the intraparticle diffusion model.

**Table 2:** Parameters for the three isotherm models

Isotherm models	Parameters	
Langmuir	*Q*_0_ (mg/g)	719.42
	*b* (L/mg)	0.057
	*R* ^2^	0.8536
Freundlich	*K*_F_ [mg/g (L/mg)]^1/*n*^	53.44
	*n*	1.13
	*R* ^2^	0.9210
Temkin	*A* (L/g)	1.66
	*B*	99.88
	*R* ^2^	0.9894

### Studies of photocatalytic activity

**Fig. 11:**
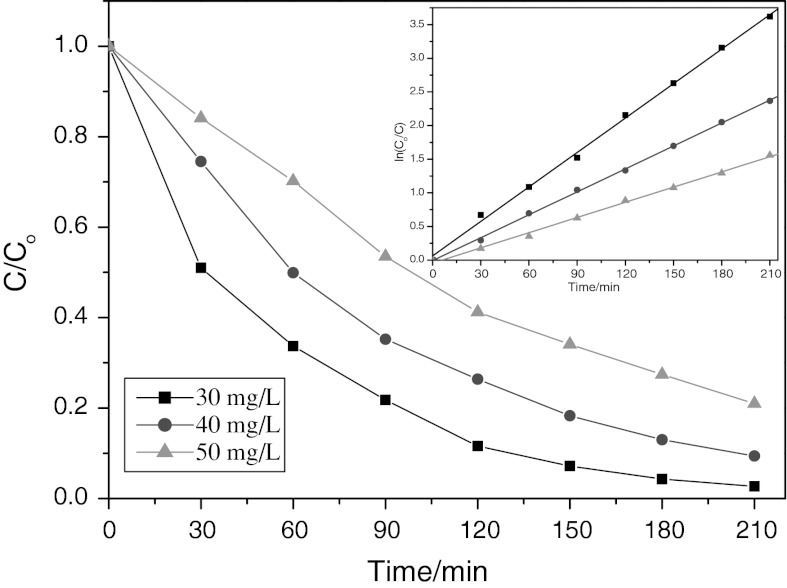
Effect of irradiation time on photodegradation of CR with different initial concentration. *Inset* presents the degradation kinetics of CR by means of plotting ln(*C*_0_/*C*) vs. time. Volume = 200 mL, amount of NiS NPs = 0.5g/L, initial pH (7.2) was used

Figure 11 displays the photodegradation of CR over NiS NPs as a function of CR concentration versus irradiation time under visible-light irradiation. For different concentration of original CR aqueous solution, the photocatalytic degradation rate of CR is quite different after 210 min illumination. For CR concentration of 30 mg/L, the degradation was almost completed after illumination for 210 min. However, for CR concentration of 50 mg/L, only about 80 % of CR was degraded at this time. Therefore, it seemed that the photodegradation efficiency of CR photocatalyzed by the NiS NPs powder decreased along with increasing original CR concentration. The decrease of the degradation efficiency of the semiconductor with the increase of CR concentration is mainly caused by two reasons. On the one hand, more CR molecules will be adsorbed on the surface of the photocatalysts with increasing concentration of CR and the active sites of the catalysts will be diminished. Therefore, the generation of hydroxyl radicals will be decreased with incremental occupied space of catalyst surface. On the other hand, increasing amounts of CR can result in decreasing the number of photons which arrived to the surface of catalysts. The light is adsorbed by CR molecules and the excitation of photocatalyst particles by photons will be diminished. Therefore, the efficiency of photodegradation decreased.

In order to determine the kinetics of photodegradation, the relationship between ln*C*_0_/*C*_t_ and irradiation time was also plotted (see Fig. 11, inset). The linear relationship between ln(*C*_0_/*C*) and time demonstrated that the photocatalytic degradation of CR followed a pseudo-first-order kinetics:

7 $$ \ln \frac{{C_{0} }}{C} = k_{1} t $$

where *C*_0_/*C* is the normalized CR concentration, *t* is the reaction time, and *k*_1_ is the reaction rate constant (min^−1^). The rate constant was calculated to be 0.0171, 0.0114, and 0.0042 min^−1^ for the CR initial concentration of 30, 40, and 50 mg/L, respectively.

**Fig. 12:**
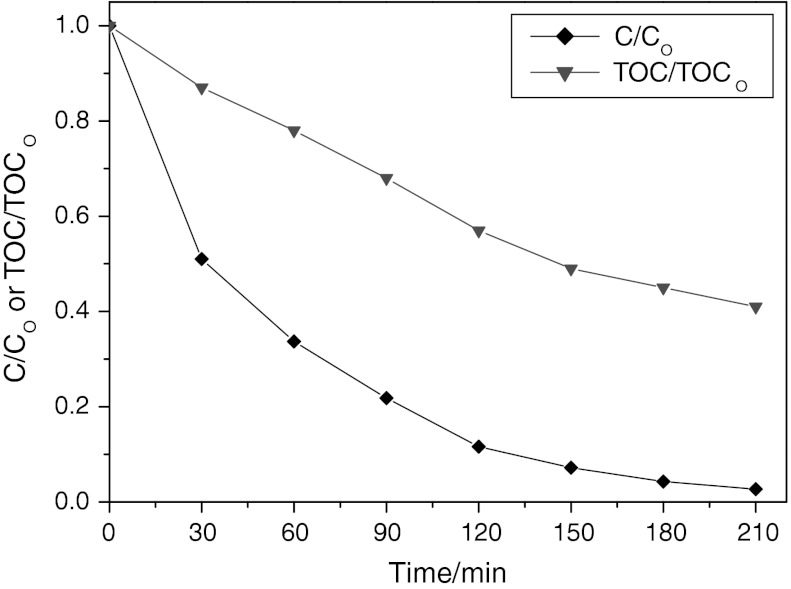
The degradation and TOC removal of the CR solution under visible-light illumination. Volume = 200 mL, [CR] = 30 mg/L, amount of nano-NiS = 0.5 g/L, initial pH was used

TOC removal of the CR solution has been also studied under the same condition. As shown in Fig. 12, after a period of 210 min, 98 % of CR was degraded while only 40.6 % of TOC of the CR solution was removed. Clearly, the TOC removal of the CR solution was lower than the degradation of CR, suggesting that the intermediates occurred during the photocatalytic process.

The determination of •OH radical’s formation in the process of photocatalytic degradation under visible-light irradiation was indirectly detected by fluorescence technique using terephthalic acid (TA), which readily reacted with •OH radicals to produce highly fluorescent product, 2-hydroxyterephthalic acid (Li et al. 2010). The feasibility of the detection is based on the fact that the intensity of the peak attributed to 2-hydroxyterephthalic acid was known to be proportional to the amount of •OH radicals formed.

**Fig. 13:**
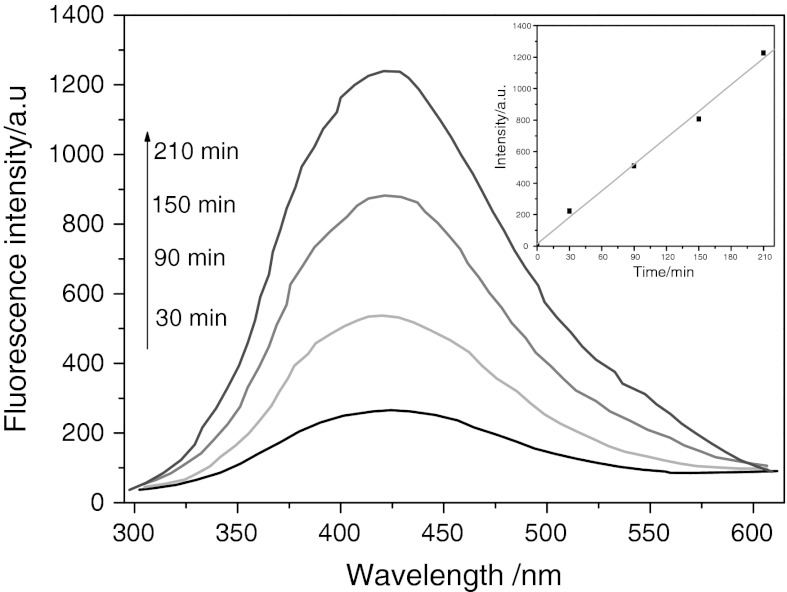
OH trapping PL spectra of NiS nanoparticle on TA solution under visible irradiation. *Inset* presents the plot from fluorescence intensity versus time

The fluorescence emission spectrum (excitation at 315 nm) of TA solution in the presence of NiS NPs powders was measured every 60 min during illumination. Figure 13 showed the induction of fluorescence from 5 × 10^−4^ M TA solution in 2 × 10^−3^ M NaOH. As shown in the figure, gradual increase in the fluorescence intensity at about 425 nm was observed with increasing illumination time, indicating that the photogenerated O_2_^−^, HO•, and H_2_O_2_ did not interfere with the reaction between •OH and TA (Xiao and Ouyang 2009). Moreover, the generated spectrum had the identical shape and maximum wavelength with that of 2-hydroxyterephthalic acid. These results suggested that fluorescent products formed from the specific reaction between •OH radicals and TA in the presence of NiS NPs under visible light.

The plot of increase in fluorescence intensity against illumination time at 425 nm has been also discussed (Fig. 13, inset). The fluorescence intensity by visible-light illumination in TA solutions increased almost linearly against time. Consequently, it can be concluded that the •OH radicals formed at the NiS NPs interfaces were in proportion to the light illumination time obeying zero-order reaction rate kinetics.

## Conclusions

1. The NiS NPs with efficient adsorption and visible-light-driven photocatalytic properties has been successfully prepared via hydrothermal method.
2. The adsorption kinetics was evaluated by the pseudo-first-order, pseudo-second-order, and Weber’s intraparticle diffusion model. The pseudo-second-order model was the best to describe the adsorption kinetics, and intraparticle diffusion was not the rate-limiting step.
3. The equilibrium adsorption data were analyzed with three isotherm models (Langmuir model, Freundlich model, and Temkin model). The best agreement was achieved by the Temkin isotherm with correlation coefficient of 0.989.
4. The as-prepared NiS NPs exhibit efficient photocatalytic activity in the degradation of Congo red under visible-light irradiation. The photocatalytic degradation of CR followed a pseudo-first-order kinetics.
5. The •OH radicals, as a main oxidant species, formed in the process of photocatalytic degradation were detected through fluorescence technique.
